# Risk of ischemic stroke in patients with ovarian cancer: a nationwide population-based study

**DOI:** 10.1186/1741-7015-12-53

**Published:** 2014-03-25

**Authors:** Ai-Seon Kuan, Chung-Jen Teng, Hua-Hsi Wu, Vincent Yi-Fong Su, Yung-Tai Chen, Sheng-Hsuan Chien, Chiu-Mei Yeh, Li-Yu Hu, Tzeng-Ji Chen, Cheng-Hwai Tzeng, Chia-Jen Liu

**Affiliations:** 1 Department of Neurosurgery, Neurological Institute, Taipei Veterans General Hospital, Taipei, Taiwan; 2 School of Medicine, National Yang-Ming University, Taipei, Taiwan; 3 Institute of Public Health, National Yang-Ming University, Taipei, Taiwan; 4 Division of Oncology and Hematology, Department of Medicine, Far Eastern Memorial Hospital, New Taipei City, Taiwan; 5 Institute of Clinical Medicine, School of Medicine, National Yang-Ming University, Taipei, Taiwan; 6 Department of Obstetrics & Gynecology, Taipei Veterans General Hospital, Taipei, Taiwan; 7 Department of Chest Medicine, Taipei Veterans General Hospital, Taipei, Taiwan; 8 Department of Medicine, Taipei City Hospital Heping Fuyou Branch, Taipei, Taiwan; 9 Division of Hematology and Oncology, Department of Medicine, Taipei Veterans General Hospital, Taipei, Taiwan; 10 Department of Family Medicine, Taipei Veterans General Hospital, Taipei, Taiwan; 11 Department of Psychiatry, Kaohsiung Veterans General Hospital, Kaohsiung, Taiwan

**Keywords:** Chemotherapy, Ischemic stroke, Ovarian cancer, Platinum, Population-based study

## Abstract

**Background:**

Cancer patients are at risk of thromboembolism. However, studies investigating the relationship between ovarian cancer and ischemic stroke are lacking. The objectives of this study were to assess the association between ovarian cancer and ischemic stroke, and to determine the predictive risk factors.

**Methods:**

Ovarian cancer patients aged 20 years and older without antecedent cerebrovascular events and who were followed up for more than 1 year between 1 January 2003 and 31 December 2011 were recruited from the Taiwan National Health Insurance database. Hazard ratios (HRs) of stroke risk for ovarian cancer patients compared with an age- and comorbidity-matched cohort were calculated by Cox proportional regression analysis. The difference in cumulative ischemic stroke incidence between ovarian cancer patients and the matched cohort was analyzed with the Kaplan-Meier method and tested with the log-rank test.

**Results:**

Each cohort (ovarian cancer and matched cohort) consisted of 8,810 individuals, with a median age of 49 years. After a median follow-up of 2.68 and 3.85 years, respectively, the ischemic stroke incidence was 1.38-fold higher in the ovarian cancer cohort than in the comparison cohort (9.4 versus 6.8 per 1,000 person-years), with an age- and comorbidity-adjusted HR of 1.49 (*P* <0.001). The ischemic stroke risk imposed by ovarian cancer was more prominent in patients under 50 years old (HR 2.28; *P* <0.001) compared with patients 50 years and older (HR 1.33; *P* = 0.005). Significant risk factors predicting stroke development were age 50 years and older (HR 2.21; *P* <0.001), hypertension (HR 1.84; *P* <0.001), diabetes mellitus (HR 1.71; *P* <0.001), and treatment with chemotherapy (HR 1.45; *P* = 0.017), especially platinum-based regimens.

**Conclusions:**

Ovarian cancer patients were at an increased risk of developing ischemic stroke. Age, hypertension, diabetes, and chemotherapy treatment were independent risk factors.

## Background

Ovarian cancer is the most common cause of cancer death from gynecologic tumors and is an important global issue requiring further attention [1]. Each year, more than 225,000 new ovarian cancers are diagnosed, and more than 140,000 deaths caused by ovarian cancer are estimated to occur worldwide [2]. The introductions of surgical cytoreduction, platinum-taxane-based chemotherapy, and, most recently, intraperitoneal chemotherapy have significantly increased life expectancy with ovarian cancer. Consequently, the median survival for ovarian cancer patients who undergo optimal surgery and adjuvant chemotherapy can be as high as 60 to 110 months [3].

However, the occurrence of comorbidities, such as cerebrovascular complications, after the cancer event may exacerbate mortality in cancer survivors. In addition, the median survival after stroke in cancer patients is 4.5 months, and treatment has no survival benefit [4]. In a large autopsy series, up to 14.6% of cancer patients had cerebrovascular complications [5]. Cancer and ischemic stroke can cause heavy burdens on the economic and healthcare systems and can disrupt the quality of life in survivors. Therefore, it is very important to identify cancer survivors who have a high risk for stroke and to provide them with close surveillance.

In ovarian cancer patients, there are a few studies and reports mentioning the occurrence of stroke [6,7]. However, no large-scale study has examined the association of ovarian cancer and ischemic stroke. Hence, it remains unknown whether ovarian cancer patients exhibit an elevated risk of developing ischemic stroke compared with the general population. Therefore, a population-based cohort study using the Taiwan National Health Insurance (NHI) was conducted to examine this issue.

## Methods

### Data sources

Taiwan’s NHI program is a universal healthcare program that was implemented in 1995. It currently covers more than 99.5% of the entire population [8]. Data used in this study were obtained from the research database of NHI, which contains encrypted computerized medical claims, inpatient and ambulatory care records, data from the Registry for Catastrophic Illness Patients (RCIP), basic demographic information, and other healthcare data. The accuracy of diagnoses in this database has been validated for several diseases, including ischemic stroke [9]. Diseases were coded with the cancer International Classification of Diseases, Ninth Revision, Clinical Modification (ICD-9-CM) diagnosis codes, 2001 edition. Because the Taiwan NHI research database contains encrypted computerized data for research purposes, the ethics committee of Taipei Veterans General Hospital informed us that this study was exempt from full review and that informed consent from each patient was not required.

### Study subjects

Two cohorts, an ovarian cancer cohort and a matched control cohort, were enrolled in this study. The ovarian cancer cohort (ICD-9-CM code 183.X) was identified from the RCIP. For a patient to be enrolled in the RCIP, the diagnosis of ovarian cancer had to be confirmed by histology. The diagnosis was subject to periodic review by the Bureau of NHI. Patients with a new ovarian cancer diagnosis made between 1 January 2003 and 31 December 2011 were included in this study. Subjects younger than 20 years old, those who had been diagnosed with any cerebrovascular disease before ovarian cancer diagnosis, and those who were lost to follow-up were excluded. Each individual in the ovarian cancer group was matched on the basis of age, gender, time of enrollment, and comorbidities related to cerebrovascular events with an individual without ovarian cancer in a comparison cohort, which was also selected from the NHI.

### Outcome measures

Occurrence of ischemic stroke was identified after the initial diagnosis of ovarian cancer using medical claims data in the NHI. Ischemic stroke identification using data in the Taiwan NHI is highly accurate compared to other insurance databases and is valid for population-based research [9]. In our study, identification was made on the basis of ischemic stroke coding (ICD-9-CM code 436, 433.X, 434.X, and 437.1X) accompanied by computed tomographic or magnetic resonance images. Diagnoses lacking confirmatory image were not included. Subjects in both cohorts (ovarian cancer and matched) were followed until a diagnosis of ischemic stroke was made, death occurred, 31 December 2011 was reached, or the patient withdrew from the NHI.

### Statistical analyses

Microsoft SQL Server 2012 (Microsoft Corporation, Redmond, WA, USA) and SAS 9.2 software (SAS Institute Inc., Cary, NC, USA) were used for data management and analysis. All statistical analyses were performed using SPSS statistical software version 17.0 for Window (SPSS, Inc., Chicago, IL, USA). Statistical significance was defined as a *P* value less than 0.05.

The demographic and clinical characteristics of ovarian cancer patients and matched cohorts are presented as the total number (n) and proportion (%). Continuous and categorical variables were compared between groups with the Mann-Whitney *U* test and Pearson’s chi-squared test, as appropriate. To assess the effect of age, study subjects were divided into two subgroups: age <50 years and age ≥50 years. Hazard ratio (HR) and 95% confidence intervals (CI) were calculated with the Cox proportional hazard models to test the association between ovarian cancer and ischemic stroke; the HR was further adjusted for age and comorbidities. The difference in the cumulative incidence of ischemic stroke between the ovarian cancer patient cohort and the matched cohort was calculated with the Kaplan-Meier method and tested with the log-rank test.

Conventional risk factors for stroke, such as diabetes mellitus and hypertension, which are also possible confounding factors, were further assessed for their effects on the association of ovarian cancer and subsequent ischemic stroke in univariate Cox proportion hazards models. Additionally, therapeutic modalities, such as surgery and chemotherapy, were put into Cox models as time-dependent covariates to avoid immortal time bias. All risk factors with *P* values <0.1 in the univariate model were further entered into the multivariate analysis.

## Results

### Clinical characteristics of the study population

**Figure 1:**
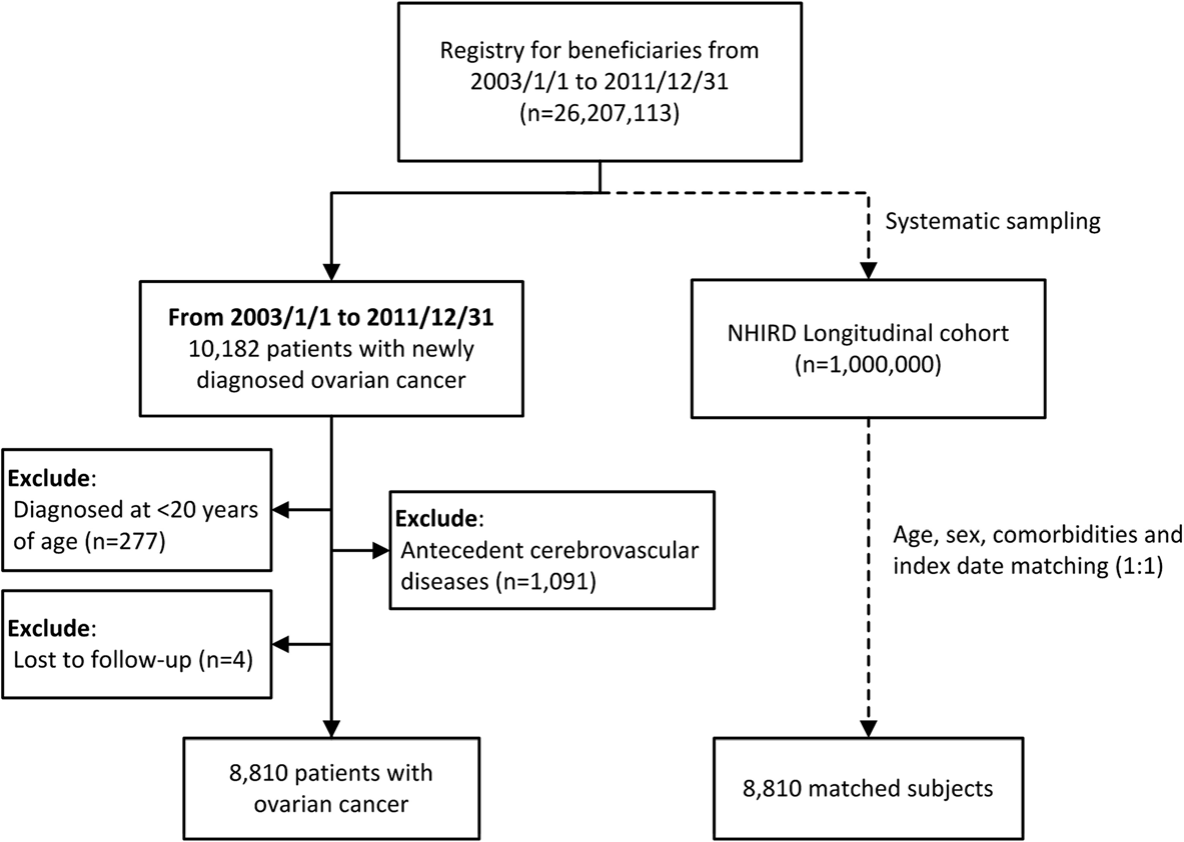
Participant selection flow chart.

A total of 10,182 patients with ovarian cancer diagnosed between 1 January 2003 and 31 December 2011 were identified. Patients who were younger than 20 years old (n = 277), diagnosed with cerebrovascular disease before ovarian cancer diagnosis (n = 1,091), or lost to follow-up (n = 4) were excluded. In total, 8,810 ovarian cancer patients were included in the study (Figure 1). The demographic characteristics of the study group and matched cohort (1:1 ratio of patient number) are shown in Table 1.

**Table 1:** Baseline patient characteristics of subjects with and without ovarian cancer

**Demographic data**	**Subjects with ovarian cancer**		**Subjects without ovarian cancer**		***P***
	**n = 8,810**		**n = 8,810**		
	**n**	**%**	**n**	**%**	
**Age (interquartile range)**	49 (41 to 58)		49 (41 to 58)		1.000
≥50 years	4,357	49.5	4,357	49.5	1.000
<50 years	4,453	50.5	4,453	50.5	
**Comorbidities**					
Diabetes mellitus	1,446	16.4	1,447	16.4	0.984
Hypertension	2,425	27.5	2,426	27.5	0.987
Chronic kidney disease	709	8.0	708	8.0	0.978
Dyslipidemia	1,987	22.6	1,986	22.5	0.986
Coronary artery disease	52	0.6	50	0.6	0.843
Atrial fibrillation	47	0.5	45	0.5	0.834
Peripheral arterial occlusive disease	19	0.2	17	0.2	0.739
**Treatment**					
Surgery	6,160	69.9			
Chemotherapy	6,590	74.8			
Cisplatin-based	3,093	35.1			
Carboplatin-based	5,041	57.2			
Non-platinum-based	402	4.6			

All subjects in both groups were women with similar age and comorbidity distributions. The median age was 49 years for both cohorts (interquartile range (IQR), 41 to 58 years). The most common comorbidity was hypertension (27.5%). There was no difference in cerebrovascular event-related comorbidities between the two groups. In the ovarian cancer cohort, 6,160 patients (69.9%) received surgery, and 6,590 patients (74.8%) received chemotherapy.

### Incidence of ischemic stroke and risk factors

**Figure 2:**
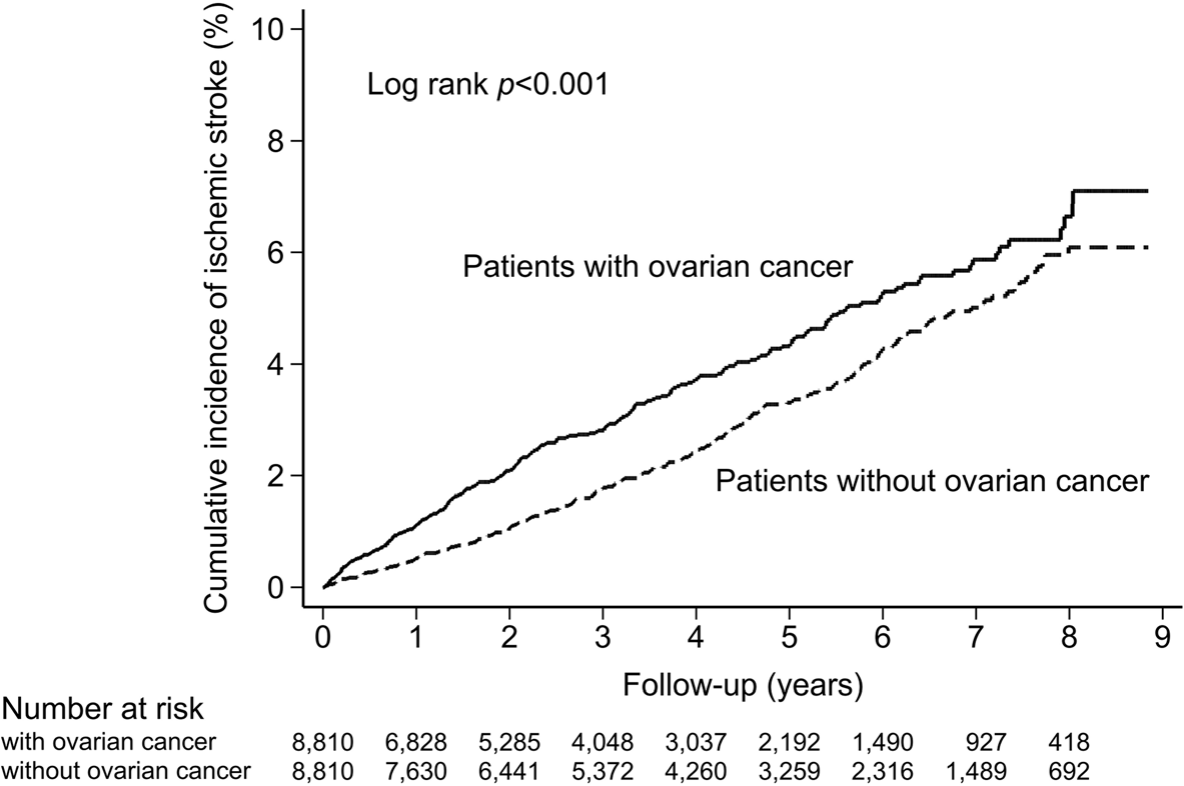
Cumulative incidence of ischemic stroke in subjects with and without ovarian cancer.

The median follow-up duration was 2.68 years (IQR, 1.44 to 4.98 years) for the ovarian cancer group and 3.85 years (IQR, 1.83 to 6.14 years) for the matched cohort group. During the follow-up period, there were 267 (9.4 per 1,000 person-years) new onset ischemic strokes in the study cohort and 244 (6.8 per 1,000 person-years) in the matched cohort. The incidence of ischemic stroke after the time of enrollment increased with age in both cohorts. The log-rank test showed a higher cumulative incidence of ischemic stroke in the ovarian cancer group than in the matched cohort (*P* <0.001) (Figure 2).

Compared with the matched cohort, the crude HR for subsequent ischemic stroke after ovarian cancer was 1.38 (95% CI, 1.16 to 1.64; *P* <0.001). After adjusting for age and comorbidities, the HR was 1.49 (95% CI, 1.25 to 1.78; *P* <0.001). When the study subjects were stratified into two subgroups, the relative risk (RR) of stroke was higher (*P* <0.05) in subjects who were <50 years (adjusted HR 2.28; 95% CI, 1.55 to 3.36; *P* <0.001) compared with those who were ≥50 years old (adjusted HR 1.33; 95% CI, 1.09 to 1.62; *P* = 0.005) (Table 2).

**Table 2:** Incidence of ischemic stroke in subjects with and without ovarian cancer

	**Patients with ovarian cancer**		**Patients without ovarian cancer**		**Crude HR (95% CI)**	***P***	**Adjusted HR**^**a **^**(95% CI)**	***P***
	**Number of ischemic stroke**	**Per 1,000 person-years**	**Number of ischemic stroke**	**Per 1,000 person-years**				
Total	267	9.4	244	6.8	1.38 (1.16 to 1.64)	<0.001	1.49 (1.25 to 1.78)	<0.001
Age								
≥50 years	192	15.5	205	12.1	1.29 (1.06 to 1.57)	0.012	1.33 (1.09 to 1.62)	0.005
<50 years	75	4.7	39	2.1	2.27 (1.54 to 3.34)	<0.001	2.28 (1.55 to 3.36)	<0.001

^a^Adjusted for age, sex, and comorbidities. CI, confidence interval; HR, hazard ratio.

In the multivariate Cox proportion hazards model, factors independently determining the risk of subsequent ischemic stroke in ovarian cancer patients included age ≥50 years (HR 2.21; 95% CI, 1.64 to 2.99; *P* <0.001), diabetes mellitus (HR 1.71; 95% CI, 1.27 to 2.29; *P* <0.001), hypertension (HR 1.84; 95% CI, 1.39 to 2.43; *P* <0.001), and chemotherapy treatment (HR 1.45; 95% CI, 1.07 to 1.97; *P* = 0.017) (Table 3). Similarly, age ≥50 years, diabetes mellitus, and hypertension were also independent risk factors for ischemic stroke in the control cohort (Additional file 1: Table S1). Comparing different chemotherapy modalities, cisplatin-based (HR 1.38; 95% CI, 1.07 to 1.76; *P* = 0.012) and carboplatin-based regimens (HR 1.46; 95% CI, 1.13 to 1.89; *P* = 0.004) were independent risk factors for subsequent ischemic stroke, whereas non-platinum-based regimens (HR 1.12; 95% CI, 0.61 to 2.04; *P* = 0.722) were not. These risk factors remained significant when 16 patients who had a new diagnosis of brain metastasis (ICD-9-CM code 198.3) less than three months after the stroke event were censored for analysis, to avoid a short-term misclassification during acute brain insults (Additional file 1: Table S2 and S3).

**Table 3:** Analyses of risk factors for ischemic stroke in patients with ovarian cancer

**Predictive variables**	**Univariate analysis**		**Multivariate analysis**^**a**^	
	**HR (95% CI)**	***P***	**HR (95% CI)**	***P***
Age ≥50	3.25 (2.49 to 4.25)	<0.001	2.21(1.64 to 2.99)	<0.001
**Comorbidities**				
Diabetes mellitus	2.84 (2.19 to 3.67)	<0.001	1.71(1.27 to 2.29)	<0.001
Hypertension	3.15 (2.47 to 4.00)	<0.001	1.84(1.39 to 2.43)	<0.001
Chronic kidney disease	1.64 (1.12 to 2.39)	0.011	1.05(0.71 to 1.56)	0.791
Dyslipidemia	2.25 (1.75 to 2.89)	<0.001	1.09(0.81 to 1.46)	0.569
Coronary artery disease	3.66 (1.36 to 9.83)	0.010	2.01(0.74 to 5.42)	0.169
Atrial fibrillation	0.75 (0.11 to 5.31)	0.769		
Peripheral arterial occlusive disease	3.09 (0.43 to 22.02)	0.261		
**Treatment**^**b**^				
Surgery^c^	0.83 (0.64 to 1.06)	0.138		
Chemotherapy	1.67 (1.25 to 2.25)	0.001	1.45(1.07 to 1.97)	0.017
Cisplatin-based	1.35 (1.06 to 1.73)	0.017	1.38(1.07 to 1.76)	0.012
Carboplatin-based	1.76 (1.37 to 2.25)	<0.001	1.46(1.13 to 1.89)	0.004
Non-platinum-based	1.24 (0.68 to 2.26)	0.490	1.12(0.61 to 2.04)	0.722

^a^All factors with *P* <0.1 in univariate analyses were included in the Cox multivariate analysis; ^b^treatment was analyzed as a time-dependent covariate in the Cox regression model; ^c^surgery included unilateral or bilateral salpingo-oophorectomy, or debulking surgery. CI, confidence interval; HR, hazard ratio.

## Discussion

This is the first population-based study to determine the risk of new onset ischemic stroke after an ovarian cancer diagnosis. Our data revealed an increased risk of developing ischemic stroke with an adjusted HR of 1.49 among patients with ovarian cancer. Significant risk factors for ischemic stroke included age ≥50 years, diabetes mellitus, hypertension, and chemotherapy treatment.

Up to 40% of stroke cases in cancer are cryptogenic related, possibly induced by the underlying malignancy [10]. For cancer patients, RRs of developing venous thrombosis of four- to seven-fold have been reported compared with matched controls [11]. However, cancer is a broad disease and different types of cancer may have different risks. Generally, pancreas, brain, lung, and ovarian cancers have been associated with the higher risks of developing venous thrombosis [12]. In addition, the presence of disseminated intravascular coagulation in patients with ovarian cancer may indicate a hypercoagulative status [13]. One direct evidence for the hypercoagulative state is the frequently overexpressed tissue factor in ovarian cancer tissue, which could activate the extrinsic coagulation cascade and cause thrombolic events in ovarian cancer patients [14].

Although the temporal-causal relationship between ovarian cancer progression and hypercoagulation is unknown, high levels of coagulation factors and associated regulatory proteins have been observed in ovarian cancer patients [15,16]. Increased levels of circulating mucinous material produced by ovarian tumors might play a role in intravascular hypercoagulation and hyperviscosity [17]. Together, these findings suggest that a subgroup of ovarian cancer patients may have distinct coagulopathies, making these patients vulnerable for specific vascular events, including ischemic stroke. These findings may also explain why ovarian cancer survivors in our study, especially younger patients who had no established conventional stroke risk factors, possessed a higher risk of subsequent ischemic stroke.

The most well-known and medically important (that is, treatable) risk factors for ischemic stroke include hypertension, diabetes mellitus, dyslipidemia, smoking, and obesity [18]. Although lifestyle and obesity were not included in the present analysis, some epidemiological studies have demonstrated that hypertension and diabetes are significant risk factors for stroke in several cancers [19,20]. Our multivariate analyses revealed a similar result, with additional significant risk factors, including age ≥50 years old and chemotherapy treatment. These results suggest that conventional stroke risk factors remain important in the pathogenesis of stroke in ovarian cancer patients.

Our study showed that ovarian cancer patients receiving chemotherapy, particularly platinum-based regimens, might have an additionally increased stroke risk. This effect was insignificant in non-platinum-based regimens. In prior studies, chemotherapy-associated increased stroke was observed in patients with head-and-neck cancer [19,21], breast cancer [22], and urothelial carcinoma [23]. While the mechanism is still uncertain, some suggested pathophysiologies are associated with increased fibrinopeptide A and decreased fibrinolytic activity [24], elevated plasma von Willebrand factor [25], hypomagnesium-associated vascular spasm [26,27], endothelial injury [28,29], and mononuclear cell-mediated platelet activation [30]. Moreover, Li *et al*. demonstrated that platinum in a chemotherapy regimen, which is also commonly used for treating ovarian cancer patients, may increase the risk of ischemic stroke among cancer patients [31]. Cerebral infarction after cisplatin-based chemotherapy in ovarian cancer patients has also been reported previously [32,33]. A meta-analysis of 38 randomized phase II and III trials showed that cancer patients who received cisplatin-based chemotherapy demonstrated a dose-dependently increased risk (RR 1.67, 95% CI, 1.25 to 2.23; *P* = 0.01) of thromboembolism compared with patients who received a non-cisplatin-based regimen [34]. More research is needed to clarify the role of chemotherapy in the relationship of ovarian cancer and subsequent ischemic stroke, as well as to determine whether it is necessary to use a prophylactic antiplatelet agent in high-risk patients during the chemotherapy period.

Our study revealed that ovarian cancer patients appeared to have a higher risk of stroke soon after cancer diagnosis, and the increased risk persisted throughout the follow-up period. A similar phenomenon was also observed in survivors of head-and-neck cancer [19,21], cervical cancer [35], breast cancer [36], and Hodgkin lymphoma [37]. The stroke risk imposed by ovarian cancer seemed to be more prominent in people of younger age, with adjusted HRs of 2.28 and 1.33 in the age groups of <50 years and ≥50 years, respectively. These findings suggest that, with increasing age, the age factor becomes more important than cancer as a risk factor of stroke.

This study has several limitations. First, lifestyle variables and behavioral factors, such as tobacco and alcohol use, body mass index, dietary habits, and biochemistry profiles including serum D-dimer level and disseminated intravascular coagulation profiles, were not available in the claims data of the Taiwan NHI. Second, cancer staging and histology were not available in this database, and their association with ischemic stroke could not be identified. Finally, information on the cause of death was not available; consequently, we cannot determine the impact of stroke on cancer-related mortality. Despite these limitations, our study was based on a nationwide, population-based database that could identify all cases of ovarian cancer and ischemic stroke in the study period. The large sample size in our study contributed to substantial statistical power and revealed a clear association between ovarian cancer and ischemic stroke, with subtle statistically significant differences between the two cohorts.

## Conclusions

This nationwide population-based study demonstrated that ovarian cancer patients are at an increased risk of subsequent ischemic stroke. Significant risk factors included age ≥50 years, hypertension, diabetes mellitus, and chemotherapy treatment, especially platinum-based regimens, following a diagnosis of ovarian cancer. With the longer expected life span of ovarian cancer patients, close surveillance and proper stroke-prevention strategies may be needed. Further prospective clinical studies of the relationship between ovarian cancer and ischemic stroke are warranted.

## Abbreviations

CI: confidence interval; HR: hazard ratio; ICD-9-CM: International Classification of Diseases, Ninth Revision, Clinical Modification; IQR: interquartile range; NHI: National Health Insurance; RCIP: Registry for Catastrophic Illness Patients; RR: relative risk.

## Competing interests

The authors declare that they have no competing interests.

## Authors’ contributions

ASK and CJL had full access to all of the data in the study and take responsibility for the integrity of the data and the accuracy of the data analysis. ASK, CJT, and CJL designed the study. CMY and CJL acquired the data and performed statistical analysis. ASK, CJT, HHW, TJC, and CHT gave the final interpretation of the results. ASK and CJT drafted the manuscript. CJT, HHW, VYS, YTC, SHC, and LYH gave critical revision of the manuscript for important intellectual content. CMY, TJC and CHT gave administrative, technical, and material support. TJC, CHT and CJL were the study supervisors. CJL acts as guarantor and accepts responsibility for the integrity of the work as a whole. All authors read and approved the final manuscript.

## Pre-publication history

The pre-publication history for this paper can be accessed here:

http://www.biomedcentral.com/1741-7015/12/53/prepub

## Supplementary Material

Additional file 1: Table S1 Analyses of risk factors for ischemic stroke in patients without ovarian cancer. **Table S2.** Incidence of ischemic stroke in patients with and without ovarian cancer (excluding those with brain metastasis within 3 months after stroke). **Table S3.** Analyses of risk factors for ischemic stroke in patients with ovarian cancer (excluding those with brain metastasis less than 3 months after a stroke). Click here for file
