# Supplementary material for: Risk of ischemic stroke in patients with ovarian cancer: a nationwide population-based study
Source: BMC Med. 2014 Mar 25;12:53. doi: 10.1186/1741-7015-12-53 (PMC4022213; doi:10.1186/1741-7015-12-53)
Supplement: Additional file 1: Table S1 — Analyses of risk factors for ischemic stroke in patients without ovarian cancer. Table S2. Incidence of ischemic stroke in patients with and without ovarian cancer (excluding those with brain metastasis within 3 months after stroke). Table S3. Analyses of risk factors for ischemic stroke in patients with ovarian cancer (excluding those with brain metastasis less than 3 months after a stroke). [file 1741-7015-12-53-S1.docx]

Additional table 1 Analyses of risk factors for ischemic stroke in patients without ovarian cancer

| **Predictive variables** | **Univariate analysis** | | **Multivariate analysis^a^** | |
| --- | --- | --- | --- | --- |
|  | **HR (95% CI)** | ***P* value** | **HR (95% CI)** | ***P* value** |
| Age ≥50 | 5.99 (4.25–8.43) | <0.001 | 3.89 (2.69–5.62) | <0.001 |
| Comorbidities |  |  |  |  |
| Diabetes mellitus | 2.76 (2.11–3.60) | <0.001 | 1.53 (1.14–2.06) | 0.005 |
| Hypertension | 4.39 (3.40–5.67) | <0.001 | 2.43 (1.82–3.25) | <0.001 |
| Chronic kidney disease | 1.55 (1.04–2.29) | 0.030 | 0.93 (0.62–1.40) | 0.739 |
| Dyslipidemia | 2.11 (1.62–2.74) | <0.001 | 0.88 (0.66–1.19) | 0.413 |
| Coronary artery disease | 2.87 (1.07–7.71) | 0.037 | 1.33 (0.49–3.59) | 0.576 |
| Atrial fibrillation | 4.74 (2.11–10.65) | <0.001 | 2.02 (0.89–4.59) | 0.091 |
| Peripheral arterial occlusive disease | 2.54 (0.36–18.13) | 0.352 |  |  |

HR, hazard ratio; CI, confidence interval.

^a^All factors with *P* < 0.1 in univariate analyses were included in the Cox multivariate analysis.

Additional table 2 Incidence of ischemic stroke in patients with and without ovarian cancer (excluding those with brain metastasis within 3 months after stroke)

|  | **Patients with**  **ovarian cancer** | |  | **Patients without**  **ovarian cancer** | | **Crude HR**  **(95% CI)** | ***P* value** | **Adjusted HR***  **(95% CI)** | ***P* value** |
| --- | --- | --- | --- | --- | --- | --- | --- | --- | --- |
|  | **No. of ischemic stroke** | **Per 1,000 person-years** |  | **No. of ischemic stroke** | **Per 1,000 person-years** |  |  |  |  |
| Total | 251 | 8.8 |  | 245 | 6.9 | 1.29  (1.08–1.54) | 0.005 | 1.40  (1.17–1.67) | <0.001 |
| Age |  |  |  |  |  |  |  |  |  |
| ≥50 | 182 | 14.7 |  | 206 | 12.2 | 1.22  (1.00–1.49) | 0.051 | 1.26  (1.03–1.54) | 0.023 |
| <50 | 69 | 4.3 |  | 39 | 2.1 | 2.08  (1.40–3.08) | <0.001 | 2.09  (1.41–3.10) | <0.001 |

HR, hazard ratio; CI, confidence interval.

* Adjusted for age, sex, and comorbidities including diabetes mellitus, hypertension, chronic kidney disease, dyslipidemia, coronary artery disease, atrial fibrillation, and peripheral arterial occlusive disease.

Additional table 3 Analyses of risk factors for ischemic stroke in patients with ovarian cancer (excluding those with brain metastasis within 3 months after stroke)

| **Predictive variables** | **Univariate analysis** | | **Multivariate analysis^a^** | |
| --- | --- | --- | --- | --- |
|  | **HR (95% CI)** | ***P* value** | **HR (95% CI)** | ***P* value** |
| Age ≥50 | 3.35 (2.53–4.42) | <.0001 | 2.19 (1.61–2.98) | <.0001 |
| **Comorbidities** |  |  |  |  |
| Diabetes mellitus | 3.00 (2.30–3.90) | <.0001 | 1.81 (1.34–2.44) | 0.000 |
| Hypertension | 3.29 (2.57–4.22) | <.0001 | 1.90 (1.43–2.53) | <.0001 |
| Chronic kidney disease | 1.69 (1.15–2.48) | 0.008 | 1.07 (0.72–1.59) | 0.745 |
| Dyslipidemia | 2.20 (1.69–2.85) | <.0001 | 1.02 (0.76–1.37) | 0.907 |
| Coronary artery disease | 3.90 (1.45–10.47) | 0.007 | 1.95 (0.72–5.29) | 0.188 |
| Atrial fibrillation | 0.80 (0.11–5.66) | 0.819 |  |  |
| PAOD | 3.27 (0.46–23.30) | 0.236 |  |  |
| **Treatment^b^** |  |  |  |  |
| Surgery | 0.77 (0.59–0.99) | 0.042 |  |  |
| Chemotherapy | 1.55 (1.15–2.08) | 0.004 | 0.68 (0.41–1.14) | 0.142 |
| Cisplatin-based | 1.34 (1.03–1.72) | 0.026 | 1.63 (1.18–2.26) | 0.003 |
| Carboplatin-based | 1.62 (1.26–2.09) | 0.000 | 1.70 (1.14–2.51) | 0.009 |
| Non-platinum-based | 1.32 (0.72–2.41) | 0.369 | 1.49 (0.79–2.80) | 0.216 |

HR, hazard ratio; CI, confidence interval; PAOD, peripheral arterial occlusive disease.

^a^All factors with *P* < 0.1 in univariate analyses were included in the Cox multivariate analysis.

^b^Treatment was analyzed as a time-dependent covariate in the Cox regression model.
